# A Questionnaire Survey of Management of Patients with Aneurysmal Subarachnoid Haemorrhage in Poland

**DOI:** 10.3390/ijerph17114161

**Published:** 2020-06-11

**Authors:** Mariusz Hofman, Norbert Hajder, Izabela Duda, Łukasz J. Krzych

**Affiliations:** Department of Anaesthesiology and Intensive Care, Faculty of Medical Sciences in Katowice, Medical University of Silesia, 40-007 Katowice, Poland; norberthajder@gmail.com (N.H.); diza33@gmail.com (I.D.); lkrzych@sum.edu.pl (Ł.J.K.)

**Keywords:** intracranial aneurysm, subarachnoid hemorrhage, ultrasonography, Doppler, transcranial, embolisation, therapeutic, vasospasm, intracranial

## Abstract

Background: Aneurysmal subarachnoid haemorrhage (aSAH) remains a potentially devastating threat to the brain with a serious impact on mortality and morbidity. We attempted to investigate correspondence between the current guidelines for aSAH management and real clinical practice in Poland. Methods: A web-based questionnaire was performed between 03.2019 and 06.2019. Centres performing neuro-interventional radiology procedures and neuro-critical care were included (*n* = 29). One response from each hospital was recorded. Results: In three (10.4%) centres, there was no clear protocol for an interventional treatment plan. Endovascular embolisation was predominantly used in 11 (37.9%) hospitals, and microsurgical clipping, in 10 (34.5%). A written protocol for standard anaesthetic management was established only in six (20.7%) centres for coiling and in five (17.2%) for microsurgical clipping. The diagnosis of cerebral vasospasm was based on transcranial Doppler as the first-choice method in seven (24.1%) units. “3-H therapy” was applied by 15 (51.8%) respondents, and “2-H therapy”, by four (13.8%) respondents. In only eight (27.6%) centres were all patients with aSAH being admitted to the ICU. Conclusion: Many discrepancies exist between the available guidelines and clinical practice in aSAH treatment in Poland. Peri-procedural management is poorly standardised. Means must be undertaken to improve patient-oriented treatment and care.

## 1. Introduction

Aneurysmal subarachnoid haemorrhage (aSAH) remains a potentially devastating threat to the brain with a serious impact on the mortality, morbidity, physical outcomes, neurocognitive performance and quality of life of the affected population [1,2]. Its treatment is challenging and multifactorial. Many aspects of pharmacological and non-pharmacological management continue to be unrated in recent recommendations, and many targets of treatment are undefined by current knowledge [3,4].

In 2015, Velly and colleagues performed an international multicentre questionnaire study to determine the clinical practice of physicians treating aSAH and to evaluate any discrepancy between practice and published evidence [5]. They found striking variability amongst the surveyed, with significant differences between countries and participating hospitals. That primary analysis was extended, demonstrating different patterns of heterogeneity that existed among participating countries [5]. Importantly, the investigators included 268 physicians from 172 institutions from 12 European countries.

Till now, this interesting issue was investigated in Austria [5], France [5,6], Germany [5,7], Greece [8], Italy [5], Spain [5,9], Switzerland [5], the United Kingdom and Ireland [5,10,11], as well as in India [12], North America [13,14], Australia and New Zealand [15]. Most of the studies revealed the astonishing variety of treatment practices and advised a need for further research [5,6,7,8,9,10,11,12,13,14,15]. Setting up centre-specific operating procedures to unify aSAH management is suggested to standardise the screening, triage, monitoring and treatment of vulnerable subjects, especially in high-volume hospitals with diverse medical conceptions and attitudes towards treatment. Patient-oriented management still seems crucial to warrant acceptable prognosis.

Due to a paucity of up-to-date data, we sought to investigate the clinical practice of Polish anaesthesiologists regarding aSAH treatment, working in hospitals performing coiling and/or clipping of the aneurysm.

## 2. Materials and Methods

### 2.1. Participants

We performed a web-based questionnaire study among anaesthesiologists in Poland. The study group comprised experienced chief-physicians of Anaesthesiology and Intensive care Units, who were working in centres performing coiling and/or clipping procedures. To select study participants, 45 centres performing neuro-interventional radiology procedures and neuro-critical care were chosen from the list of all hospitals obtained from the national health provider system. The invitation for participation with an interactive link to the questionnaire was sent twice by e-mail from March to June 2019. After a failed second attempt in e-based communication, an additional two phone calls were performed to renew the invitation and to remind of the study procedures. The response rate was 64.4% (*n* = 29). Only one response given by the chief physician from each hospital regarding centre-specific procedures was recorded. Most responses were provided by anesthesiologists focusing on both neuro-anaesthesia and neuro-intensive care (*n* = 25, 86.2%).

### 2.2. Questionnaire

To make the results more comparable with the available European data, we applied a Polish version of the questionnaire developed originally in English by Velly et al. [5]. Briefly, it investigated three areas of aSAH management: anaesthetic management during coiling and clipping procedures, the approach to the diagnosis of cerebral vasospasm, and the medical and interventional management of symptomatic vasospasm. We performed a professional translation (English to Polish), reverse translation (Polish to English) and second translation (English to Polish) of the questionnaire to ensure the high quality of the data, according to the acknowledged procedures [16].

The study was voluntary and anonymous. Under sections 21 and 22 of the Act of 5 December 1996 on the Medical Profession, due to the non-interventional design of the study, no approval of the Ethics Committee was required [17].

### 2.3. Statistical Analysis

Statistical data were recorded using the licensed MedCalc version 17.2 (MedCalc Software bvba, Ostend, Belgium) statistical software. Qualitative variables were depicted as crude values and percentages.

## 3. Results

More than a half of the anaesthesiologists (*n* = 18, 62.1%) declared that the interventional treatment of cerebral aneurysm in patients with aSAH is carried out as soon as possible, even in night shifts. In three (10.4%) centres, there was no clear protocol and the delay for the procedure depended on patients’ initial clinical condition or medical staff availability.

There was a variety in the first-choice treatment method: in 11 (37.9%) hospitals, more than 60% of aSAH patients underwent endovascular embolization, while in 10 (34.5%), microsurgical clipping was performed with similar prevalence; eight (27.6%) units performed both types of interventional procedures with similar frequencies. The full characteristics are presented in Table 1.

### 3.1. Anaesthetic Management of Endovascular Embolization

A written protocol for standard anaesthetic management for patients undergoing coiling was established only in six (20.7%) centres. The main anaesthetic technique was total intravenous anaesthesia (*n* = 18, 62.1%). Fentanyl was the most commonly used opioid (*n* = 18, 62.1%), although remifentanil was available as well (*n* = 9, 31.0%). The majority of responders (*n* = 25, 86.2%) established a mean arterial pressure (MAP) target level during the procedure, most commonly at 70–80mmHg (*n* = 12, 41.4%). Norepinephrine was the first-choice method to ensure the retention of the accepted values of MAP (*n* = 23, 79.3%). Ephedrine (*n* = 3, 10.3%), dopamine (*n* = 1, 3.4%) and decreasing the depth of anaesthesia (*n* = 2, 6.9%) were less often applied.

### 3.2. Anaesthetic Management of Microsurgical Clipping

A written protocol for standard anaesthetic management existed only in five(17.2%) centres. Total intravenous anaesthesia was applied in 21 (72.4%) cases. The opioids used during anaesthesia were mainly fentanyl (*n* = 17, 58.6%) and remifentanil (*n* = 10, 34.5%). Similarly, to during endovascular embolisation, during microsurgical clipping, most of the practitioners targeted MAP (*n* = 21, 72.4%). However, the target value of MAP was ambiguous, with the same number of responders choosing ranges of 60–70 mmHg and 70–80 mmHg (*n* = 7, 24.1%). Norepinephrine was similarly the primary means to achieve the target MAP (*n* = 25, 86.2%). Most of responders (n = 20, 68.9%) did not introduce any neuroprotective technique during temporary clipping. In nine (31%) hospitals, drug-induced burst-suppression was applied. Hypothermia was not used at all. Details of the peri-procedural anaesthetic management are presented in Table 2.

### 3.3. Diagnosis of Cerebral Vasospasm after aSAH

The diagnosis of cerebral vasospasm, apart from its clinical features, most commonly was based on CT angiography (*n* = 16, 55.2%). Transcranial Doppler was the first-choice method in seven (24.1%) units. Cerebral catheter angiography (*n* = 2, 6.9%) and brain tissue oxygenation measurements (*n* = 2, 6.9%) were also used by a minority of practitioners.

### 3.4. Management of Cerebral Vasospasm after aSAH

In the case of the presence of aSAH, 15 (51.8%) respondents applied “3-H therapy”, including hypertension, hypervolemia and haemodilution. An additional four (13.8%) persons used a combination of hypervolemia (“2-H therapy”) and hypertension without haemodilution. In five (17.2%) casess hypertension alone was the treatment of choice, constituting 24 (82.8%) overall applications of hypertension by practitioners. Finally, five (17.2%) responders did not include any component of “3-H therapy” in their treatment plan. Twenty-seven (93.1%) practitioners established a MAP target level. Most commonly (n = 16, 55.2%), it was appointed at above 90mmHg. The main method of reaching the above-mentioned target was the application of norepinephrine, confirmed by all respondents. Nine (31.0%) persons combined it with a drug-induced cardiac output increase, with the use of dobutamine or epinephrine in six (20.7%) and three (10.3%) cases, respectively. As hypervolemia is a component of both 3-H and 2-H therapies, it was applied by 19 (65.6%) persons. Different amounts of fluid overload were used to achieve it: in 17 (89.4%) cases, it did not exceed 2000 mL.

As a pharmacological treatment for cerebral vasospasm, nimodipine was the first-choice agent in 28 (96.6%) cases. Statins or magnesium were also mentioned but rather as additional or second-line treatments.

In the majority of centres (*n* = 20, 69.0%), an interventional approach of treating cerebral vasospasm has not been introduced yet. Nevertheless, the intra-arterial injection of a pharmacological agent and transluminal balloon angioplasty were used in six (20.7%) and one (3.4%) hospitals, respectively. Moreover, a combination of both methods is performed in two (6.9%) centres.

### 3.5. Postoperative Care

In only eight (27.6%) centres were all patients with aSAH, clinical status aside, being admitted to the ICU. Most of hospitals adopted a strategy to admit to ICU only high-grade WFNS patients.

There was no agreement in terms of prophylactic anti-epileptic treatment: the majority (*n* = 16, 55.2%) introduced it only in patients with a history of seizures and nine (31.0%) respondents did not use it at all, while four (13.8%) used it systematically.

After the interventional management of aneurysm, in patients without cerebral vasospasm, 24 (82.8%) respondents decided to set a target for systolic blood pressure. Most commonly, it was set above 110 mmg or 120 mmHg in nine (31.0%) and 10 (34.5%) responses, respectively. Full details of the responses concerning the management of vasospasm are presented in Table 3.

## 4. Discussion

It has been 8 years since the publication of the last AHA/ASA guidelines for the management of aneurysmal subarachnoid haemorrhage [3]. Additionally, the last European Stroke Organization (ESO) Guidelines for the management of intracranial aneurysms and SAH were published in 2013 [4]. Since then, international authors have carried out many clinical studies referring to specific recommendations included in those documents [18,19,20,21,22,23,24,25,26,27]. In 2015, Velly et al. performed a survey of European practice [5]. They assessed the compliance of practitioners across the continent with AHA/ASA guidelines in patients with aSAH. Nevertheless, this study did not include Poland. Therefore, the aim of our study seems justifiable.

According to guidelines, as the risk of re-bleeding in patients with aSAH is high, urgent evaluation and treatment is recommended [3,4]. Therefore, it is disappointing that only 62.1% of the responders declared that aSAH patients undergo invasive treatment of the aneurysm, if required, as soon as possible, even at night. Over 10% of centres were staff-dependent or based the necessity of urgent treatment on clinical condition, while there were no scientific data supporting such an approach. Data suggest that more than one third of re-bleeding occurs in the first 3 h and nearly half, in 6 h from primary bleeding, and delaying treatment for 72 h or more may be disadvantageous for the patient [18]. It can be inferred on that basis that 62% of the centres in our study, as well as in 35% of the hospitals in the European survey, the undertaking of the fast treatment of aneurysm is not sufficient.

Guidelines do not directly impose the choice of method of invasive treatment. It is clear that patients amenable to both methods should undergo endovascular embolisation. On the other hand, several premises may increase consideration for microsurgical clipping [3,4]. Therefore, it is expected for the choice of treatment to be centre-dependent. Both in our study and in the European survey, there are many units that prefer endovascular or microsurgical treatment.

Anaesthetic management during aneurysm treatment appears to be poorly standardised. The existence of a written protocol declared by 20.7% and 17.2% of responders in our study for endovascular and microsurgical treatment is even lower than in the European survey (35.8% and 41.8%, respectively). Most physicians set perioperative MAP target values, but as they are not clearly specified in the ASA/AHA guidelines, it is plausible that responders did not show unanimity in that matter. The most common applied target was 70–80 mmHg during both methods of treatment. It may not be efficient, as the ESO recommends MAP to be kept at least above 90 mmHg [4]. Moreover, the ESO recommends that patients with aSAH should be monitored in an ICU regimen or neurovascular unit [4]. In our study, only 27.6% of the centres admitted all patients to ICU, in great contrast with the European survey, with 72.8%. In our study, ICU admission most commonly depended on the patient’s clinical condition.

In the 2012 guidelines, the view on so-called “3-H therapy” (Hypervolemia, Hypertension, Haemodilution) shifted. Based on accumulating literature, societies have recommended the maintenance of euvolaemia and induced hypertension [3]. This was confirmed by later studies [19]. Moreover, intravenous volume expansion for the prophylaxis or treatment of vasospasm can be considered as adverse [20]. Therefore, it is surprising that over half of the responders still use 3-H therapy and 65.6% use hypervolemia in the treatment plan. Only 17.2% of the responders showed cooperation with the guidelines in that matter. It is a comparable outcome with that of the European survey, where Velly et al. reported a 44% usage of 3-H therapy and 22%, of 2-H therapy [5]. It is unknown why 3-H therapy still has strong place in the treatment plan, despite no evidence of its efficacy (Table A1).

In this study, 93.1% of responders, compared to 92.2% in the European study, set a target for MAP in patients with cerebral vasospasm (VS). As it is not specified by the guidelines, it is not surprising that practitioners set different target values of MAP. As norepinephrine is the main agent to secure the maintenance of accepted MAP values, almost one third of responders, similarly to in the European survey, declared that they increased cardiac output as well [5]. Such conduct is not supported or contraindicated by the guidelines and requires further studies.

The pharmacological treatment of cerebral vasospasm was based on use of nimodipine in our study, as well in the European survey, in agreement with published guidelines. Of the repsonders, 3.4% declared the use of magnesium sulphate, although recent data have shown no evidence of clinical improvement [21].

The diagnosis of cerebral VS was based on CT angiography in most cases in our study. Transcranial Doppler was used only by 24% of the practitioners, despite many studies proving its effectiveness and high sensitivity [22,23]. It is recommended to monitor the development of arterial vasospasm by the ASA/AHA guidelines [3]. It is in great contrast to the European survey where almost 80% of practitioners declared its use in the screening. Similarly, the use of an interventional method for cerebral vasospasm in our study remains rather low in comparison to that in the European survey (31.0% vs. 78.7%). However, in this case, it can be excused by the low quality of the evidence to support those methods of treatment [24,25]. However, recent studies, like the work of Boulouis et al., seem to indicate that endovascular intervention in vascular vasospasm may have a favourable influence on patients’ outcomes [26].

Like in the European survey, in our study, we have found striking differences in the use of prophylaxis with anticonvulsants. There is no agreement in guidelines on that matter. The 2012 AHA/ASA guidelines state that the use of prophylactics may be considered in the immediate post-haemorrhagic period and that long-term use may be considered in patients with known risk factors of DIND only [3]. The 2013 ESO guidelines support only the use of antiepileptics in patients with clinically apparent seizures and do not support any prophylaxis [4]. Further studies showed that there is no evidence of the benefits of prophylactic anticonvulsants in aSAH patients [27]. With the lack of the unanimity of the data, it is not surprising to find different approaches in various centres.

This study has a few limitations. First of all, it has a limited number of participants, which may not be representative of the entire population in Poland. It is not avoidable, as there is a limited number of units in the country that have experience in the treatment of aSAH patients, both in rural and urban areas (nine university and 20 non-university hospitals participated in the study). The extension of the study to a wide number of centres could result in including the answers of unexperienced practitioners and bias in the data. We tried to mitigate this shortcoming by sending our electronic invitation twice by e-mail, and additional phone calls were also performed. Secondly, as in every questionnaire study, respondents may tend to respond in a preconceived manner. Comparing our results with those of the European Survey, the time distancing of both studies must be acknowledged, as the respondents in this study could be influenced by more recent data. Moreover, the questionnaire was self-filled, so the subjectivism of the given answers cannot be avoided. Much more accurate results would be obtained during a survey with the participation of a professional interviewer, but in this case, we decided to apply an e-web-based method. Finally, we failed to focus on other therapeutic methods that can be applied to potentially improve the outcomes of patients with aSAH, including external ventricular drainage with intracranial pressure measurement [28], decompressive craniectomy in the case of brain oedema [29], antithrombotic agent reversal [30] and short-term tranexamic acid usage [31]. Additionally, secondary brain damage must be avoided as far as hyponatraemia, hyperglycaemia/hypoglycaemia and anaemia control are concerned. However, none of them was investigated by the primary study of Velly et al. [5].

## 5. Conclusions

Many discrepancies still exist between the available guidelines and recent literature data, and clinical practice in aSAH treatment in Poland. While it is anticipated that differences will be revealed in the areas where guidelines are not clear or inconclusive, it is rather astonishing that issues on which there is agreement of clinical studies and different scientific societies are still neglected or ignored by medical staff. The most striking issues are among the urgency of aneurysm treatment, supplementation of “3-H therapy” and diagnosis of cerebral vasospasm. Means must be undertaken to popularise the most recent data and introduce them into everyday clinical practice, as this is crucial to improve patient-oriented treatment and care.

## Figures and Tables

**Table 1:** General treatment plan.

Variable	*n* (%)	
**Treatment of aneurysm**		
As fast as possible, even at night	18	(62.1%)
Within 24 h after admission	5	(17.2%)
Within 48 h after admission	1	(3.4%)
Within 72 h after admission	2	(6.9%)
Clinical status or staff-dependent	3	(10.4%)
**Preferred treatment method**		
Approximately the same proportion of coiling and clipping	8	(27.6%)
>60% of clipping	4	(13.8%)
>60% of coiling	8	(27.6%)
>90% of clipping	6	(20.7%)
>90% of coiling	3	(10.3%)
**Criteria for ICU admission**		
All patients after aSAH	8	(27.6%)
Only high-grade patients (WFNS 3–5)	21	(72.4%)
**Recovery and tracheal extubation after uncomplicated surgery**		
As soon as possible in most patients	16	(55.2%)
After 1–3 h delay in the PACU	6	(20.7%)
Delayed in the ICU in most patients	7	(24.1%)
**Prophylactic anti-epileptic treatment**		
Systematic	4	(13.8%)
Only in patients with a history of seizures	16	(55.2%)
No prophylactic treatment	9	(31.0%)

**Table 2:** Perioperative anaesthetic management.

Variable	Coiling		Clipping	
	*n* (%)		*n* (%)	
Inhalation anaesthesia	11	(37.9%)	8	(27.6%)
Total intravenous anaesthesia	18	(62.1%)	21	(72.4%)
**The narcotic mainly used**				
Fentanyl	18	(62.1%)	17	(58.6%)
Sufentanyl	2	(6.9%)	2	(6.9%)
Alfentanil	0	-	0	-
Remifentanil	9	(31.0%)	10	(34.5%)
**What is your blood pressure target?**				
No specific target	4	(13.8%)	8	(27.6%)
Mean Arterial Pressure 60–70 mmHg	4	(13.8%)	7	(24.1%)
Mean Arterial Pressure 70–80 mmHg	12	(41.4%)	7	(24.1%)
Mean Arterial Pressure 80–90 mmHg	5	(17.2%)	4	(13.8%)
Mean Arterial Pressure >90 mmHg	4	(13.8%)	3	(10.3%)
**Main method to increase blood pressure**				
Norepinephrine	23	(79.3%)	25	(86.2%)
Ephedrine	3	(10.3%)	3	(10.3%)
Decrease the depth of anaesthesia	2	(6.9%)	1	(3.4%)
Dopamine	1	(3.4%)	0	-
**Neuroprotective strategy during temporary clipping**				
None or exceptional			20	(68.9%)
Hypothermia			0	-
Drug-induced burst suppression			9	(31.1%)

**Table 3:** Management of vasospasm in patients with aneurysmal subarachnoid haemorrhage (aSAH).

Variable	*n* (%)	
**Management of vasospasm**		
3-H Therapy (Hypervolemia–Haemodilution–Hypertension)	15	(51.7%)
2-H Therapy (Hypervolemia–Hypertension)	4	(13.8%)
Hypertension alone	5	(17.2%)
None of the above	5	(17.2%)
**Blood pressure management in patients with vasospasm**		
No specific target	2	(6.9%)
Mean arterial pressure >80mmHg	5	(17.2%)
Mean arterial pressure >90mmHg	16	(55.2%)
Mean arterial pressure >100mmHg	4	(13.8%)
Mean arterial pressure >110mmHg	2	(6.9%)
**Which volume above standard fluid replacement?**		
1 L per day	10	(34.5%)
1–2 L per day	7	(24.1%)
2–3 L per day	1	(3.4%)
3–4 L per day	1	(3.4%)
>4 L per day	0	-
I do not use hypervolemia	10	(34.5%)
**Nonclinical diagnosis of vasospasm relies upon**		
Transcranial Doppler	7	(24.1%)
CT perfusion	0	-
CT angiography	16	(55.2%)
Conventional angiography	2	(6.9%)
Brain tissue oxygen pressure	2	(6.9%)
None of these methods	2	(6.9%)
**Interventional management of vasospasm**		
Intra-arterial vasodilator	6	(20.7%)
Cerebral angioplasty	1	(3.4%)
Both methods	2	(6.9%)
None of the above	20	(6.9%)

## Appendix A

**Table A1:** Velly et al. Anaesthetic and ICU Management of Aneurysmal Subarachnoid Haemorrhage: A Survey of European Practice.

Variable	Study	Total	France	Italy	Germany	Spain	Great Britain and Ireland	Austria	Switzerland	Others
		Velly et al. Anaesthetic and ICU Management of Aneurysmal Subarachnoid Haemorrhage: A Survey of European Practice								
	*n* = 29	*n* = 268	*n* = 79	*n* = 51	*n* = 44	*n* = 36	*n* = 24	*n* = 16	*n* = 8	*n* = 10
**Criteria for SAH admission to ICU**										
All patients after SAH	8 (28)	193 (72)	56 (71)	24 (47)	43 (98)	27 (75)	13 (54)	14 (88)	7 (88)	9 (90)
Only high-grade patients (WFNS 3 to 5)	21 (72)	75 (28)	23 (29)	27 (53)	1 (2)	9 (25)	11 (46)	2 (12)	1 (12)	1 (10)
**Delay to treat aneurysm**										
As soon as possible even at night	18 (62)	101 (38)	24 (30)	26 (51)	22 (55)	5 (14)	2 (8)	12 (75)	6 (75)	2 (20)
<24 h after admission	5 (17)	118 (44)	43 (55)	19 (37)	18 (41)	16 (44)	12 (50)	4 (25)	1 (12)	5 (50)
<48 h after admission	1 (3)	40 (15)	12 (15)	5 (10)	2 (4)	10 (28)	8 (33)	0 (0)	1 (12)	2 (20)
<72 h after admission	2 (7)	9 (3)	0 (0)	1 (2)	0 (0)	5 (14)	2 (8)	0 (0)	0 (0)	1 (10)
Anaesthetic technique for coiling										
**Written anaesthetic protocol**										
Yes	6 (21)	96 (36)	28 (35)	15 (29)	24 (54)	14 (39)	3 (12)	3 (19)	3 (37)	6 (60)
No	23 (79)	172 (64)	51 (65)	36 (71)	20 (46)	22 (61)	21 (88)	13 (81)	5 (63)	4 (40)
**Anaesthetic technique**										
Total intravenous anaesthesia	18 (62)	195 (73)	64 (81)	33 (65)	40 (91)	20 (56)	11 (46)	13 (81)	8 (100)	6 (60)
Inhalation anaesthesia	11 (38)	73 (27)	15 (19)	18 (35)	4 (9)	16 (44)	13 (54)	3 (19)	0 (0)	4 (40)
**Opioid used**										
Remifentanil	9 (31)	184 (69)	44 (57)	38 (74)	29 (66)	31 (86)	19 (79)	15 (94)	4 (50)	4 (40)
Sufentanil	2 (7)	50 (18)	32 (39)	0 (0)	12 (27)	0 (0)	0 (0)	0 (0)	2 (25)	4 (40)
Fentanyl	18 (62)	32 (12)	3 (4)	13 (26)	3 (7)	5 (14)	4 (17)	1 (6)	1 (12)	2 (20)
None	0 (0)	2 (1)	0 (0)	0 (0)	0 (0)	0 (0)	1 (4)	0 (0)	1 (12)	0 (0)
**Management of blood pressure**										
Norepinephrine	23 (80)	140 (52)	54 (68)	21 (41)	41 (93)	13 (36)	3 (12)	11 (69)	7 (88)	1 (10)
Epinephrine	0 (0)	52 (19)	12 (15)	9 (18)	0 (0)	17 (47)	2 (8)	3 (19)	1 (12)	2 (20)
Phenylephrine	0 (0)	30 (11)	12 (15)	3 (6)	0 (0)	4 (11)	4 (17)	1 (6)	0 (0)	6 (60)
Dopamine	1 (3)	11 (4)	0 (0)	10 (20)	0 (0)	1 (3)	0 (0)	1 (6)	0 (0)	0 (0)
Metaraminol	0 (0)	14 (5)	0 (0)	0 (0)	0 (0)	0 (0)	12 (50)	0 (0)	0 (0)	0 (0)
Decrease depth of anaesthesia	2 (7)	14 (5)	1 (1)	7 (14)	0 (0)	1 (3)	0 (0)	0 (0)	0 (0)	0 (0)
Other	3 (10)	7 (3)	0 (0)	1 (2)	3 (7)	0 (0)	2 (8)	0 (0)	0 (0)	0 (0)
Anaesthetic technique for clipping										
**Written protocol of anaesthesia**										
Yes	24 (83)	112 (42)	23 (29)	20 (39)	30 (68)	17 (47)	4 (17)	7 (44)	5 (62)	6 (60)
No	5 (17)	156 (58)	56 (71)	31 (61)	14 (32)	19 (53)	20 (83)	9 (56)	3 (38)	4 (40)
**Anaesthetic technique**										
Total intravenous anaesthesia	21 (72)	207 (77)	64 (81)	33 (65)	39 (76)	27 (75)	15 (63)	13 (81)	8 (100)	8 (80)
Inhalation anaesthesia	8 (28)	61 (23)	15 (19)	18 (35)	5 (24)	9 (25)	9 (37)	3 (19)	0 (0)	2 (20)
**Opioid used**										
Remifentanil	10 (34)	191 (71)	41 (52)	38 (75)	29 (66)	35 (97)	23 (96)	15 (94)	5 (63)	5 (50)
Sufentanil	2 (7)	52 (19)	36 (46)	0 (0)	12 (27)	0 (0)	0 (0)	0 (0)	2 (25)	3 (30)
Fentanyl	17 (59)	25 (9)	2 (2)	13 (2)	3 (7)	1 (3)	1 (4)	1 (6)	1 (12)	2 (20)
**Management of blood pressure**										
Norepinephrine	25 (86)	151 (56)	50 (63)	23 (45)	38 (86)	8 (22)	1 (4)	11 (69)	7 (88)	2 (20)
Epinephrine	0 (0)	46 (17)	14 (18)	7 (14)	1 (2)	22 (61)	3 (12)	4 (12)	1 (12)	2 (20)
Phenylephrine	0 (0)	31 (12)	12 (15)	3 (6)	0 (0)	3 (8)	4 (17)	3 (19)	0 (0)	5 (50)
Dopamine	0 (0)	12 (4)	0 (0)	9 (18)	0 (0)	1 (3)	1 (4)	0 (0)	0 (0)	0 (0)
Metaraminol	0 (0)	11 (4)	0 (0)	0 (0)	0 (0)	0 (0)	15(63)	0 (0)	0 (0)	0 (0)
Decrease depth of anaesthesia	1 (3)	10 (4)	2 (2)	7 (14)	2 (5)	2 (6)	0 (0)	0 (0)	0 (0)	1 (10)
Other	3 (10)	7 (3)	1 (1)	2 (4)	3 (7)	0 (0)	0 (0)	0 (0)	0 (0)	0 (0)
**Neuroprotective strategy during temporary clipping**										
Drug-induced burst suppression	9 (31)	98 (36)	20 (25)	23 (45)	14 (32)	18 (50)	8 (33)	8 (50)	1 (12)	6 (60)
Hypothermia	0 (0)	10 (4)	1 (1)	2 (4)	3 (7)	2 (6)	1 (4)	0 (0)	1 (12)	3 (30)
None or exceptional	20 (69)	160 (60)	58 (74)	26 (51)	27 (61)	15 (42)	15 (63)	8 (50)	6 (75)	1 (10)
**Recovery and tracheal extubation after uncomplicated surgery**										
As soon as possible in most patients	16 (55)	217 (81)	67 (85)	31 (61)	38 (86)	32 (89)	23 (96)	12 (75)	7 (88)	7 (70)
After a 1 to 3 h delay in PACU	6 (21)	17 (6)	2 (2)	7 (14)	3 (7)	0 (0)	0 (0)	2 (12)	0 (0)	3 (30)
Delayed in the ICU in most patients	7 (24)	34 (13)	10 (13)	13 (25)	3 (7)	4 (11)	1 (4)	2 (12)	1 (12)	0 (0)
**Drug(s) used for prevention of vasospasm**										
Nimodipine		259 (97)	79 (100)	51 (00)	40 (91)	36 (100)	23 (96)	14 (88)	7 (88)	9 (90)
Statins		55 (20)	35 (44)	9 (18)	4 (9)	1 (3)	2 (8)	1 (6)	1 (12)	1 (10)
Magnesium		52 (19)	18 (23)	11 (22)	11 (25)	2 (6)	4 (17)	7 (44)	1 (12)	2 (20)
Nicardipine if hypertensive		13 (5)	11 (14)	1 (2)	0 (0)	0 (0)	0 (0)	1 (6)	0 (0)	0 (0)
None of the above		8 (3)	0 (0)	0 (0)	4 (7)	0 (0)	1 (4)	1 (6)	1 (12)	1 (10)
**Method(s) used for diagnosis of vasospasm**										
Transcranial Doppler	7 (24)	210 (78)	72 (94)	42 (82)	40 (91)	23 (64)	11 (46)	13 (81)	5 (62)	2 (20)
CT angiography	16 (55)	111 (41)	34 (43)	10 (20)	14 (32)	14 (39)	13 (54)	9 (56)	6 (75)	5 (50)
Conventional angiography	2 (7)	99 (37)	36 (46)	21 (41)	13 (29)	7 (19)	9 (37)	5 (31)	4 (50)	3 (30)
CT perfusion	0 (0)	72 (27)	37 (47)	10 (20)	7 (16)	5 (14)	4 (17)	4 (25)	3 (37)	2 (20)
Brain tissue oxygen tension	2(7)	24 (9)	11 (14)	0 (0)	6 (14)	1 (3)	1 (4)	2 (12)	1 (12)	0 (0)
None of the above	2 (7)	10 (4)	0 (0)	0 (0)	0 (0)	3 (8)	5 (21)	2 (12)	0 (0)	0 (0)
Medical management of symptomatic cerebral vasospasm										
**Technique used**										
“Triple-H” therapy	15 (52)	117 (44)	20 (25)	21 (41)	22 (50)	25 (70)	12 (50)	9 (56)	4 (50)	4 (40)
“Double-H” therapy	4 (14)	58 (22)	24 (30)	14(27)	10 (23)	3 (8)	4 (17)	2 (12)	0 (0)	1 (10)
Hypertension	5 (17)	80 (30)	30 (38)	14 (27)	11 (25)	5 (14)	8 (33)	3 (19)	4 (50)	5 (50)
None of the above	5 (17)	13 (5)	5 (6)	2 (4)	1 (2)	3 (8)	0 (0)	2 (12)	0 (0)	0 (0)
**Blood pressure target**										
MAP >110 mmHg	2 (7)	71 (27)	16 (23)	11 (22)	10 (23)	8 (22)	3 (13)	16 (100)	4 (50)	3 (30)
MAP >100 mmHg	4 (14)	79 (30)	33 (42)	18 (35)	11 (25)	6 (17)	7 (29)	0 (0)	2 (25)	2 (20)
MAP >90 mmHg	16 (55)	83 (31)	24 (30)	17 (33)	22 (50)	12 (33)	5 (21)	0 (0)	0 (0)	3 (30)
MAP >80 mmHg	5 (17)	15 (5)	4 (5)	2 (4)	0 (0)	6 (17)	1 (4)	0 (0)	1 (12)	1 (10)
No specific target	2 (7)	21 (7)	2 (2)	3 (6)	1 (2)	4 (11)	8 (33)	0 (0)	1 (12)	1 (10)
**Main method to increase blood pressure**										
Norepinephrine	29 (100)	241 (90)	75 (95)	41 (80)	44 (100)	30 (83)	21 (88)	16 (100)	8 (100)	7 (70)
Phenylephrine	0 (0)	9 (3)	3 (4)	0 (0)	0 (0)	3 (8)	2 (8)	0 (0)	0 (0)	1 (10)
Dopamine	0 (0)	13 (5)	0 (0)	9 (18)	0 (0)	3 (8)	0 (0)	0 (0)	0 (0)	1(10)
Others	0 (0)	5 (2)	1 (1)	1 (2)	0 (0)	0 (0)	1 (4)	0 (0)	0 (0)	1 (10)
**Fluid volume administered daily in addition to standard fluid replacement**										
No hypervolaemia	10 (35)	63 (23)	21 (27)	13 (25)	14 (32)	3 (8)	5 (21)	3 (19)	3 (37)	1 (10)
1 L	10 (35)	35 (13)	7 (9)	11 (22)	5 (11)	2 (6)	3 (12)	4 (25)	1 (12)	2 (20)
1 to 2 L	7 (24)	74 (28)	19 (24)	15 (29)	17 (39)	8 (22)	6 (25)	6 (37)	3 (37)	0 (0)
2 to 3 L	1 (3)	76 (28)	28 (35)	7 (14)	7 (16)	18 (50)	7 (29)	3 (19)	1 (12)	5 (50)
3 to 4 L	1 (3)	19 (7)	4 (5)	5 (10)	1 (2)	4 (11)	3 (12)	0 (0)	0 (0)	2 (20)
>4 L	0 (0)	1 (1)	0 (0)	0 (0)	0 (0)	1 (3)	0 (0)	0 (0)	0 (0)	0 (0)
**Management of cardiac output**										
No intervention	20 (69)	208 (78)	60 (76)	37 (72)	32 (73)	30 (83)	23 (96)	12 (75)	5 (63)	9 (90)
Dobutamine	6 (21)	47 (17)	10 (13)	12 (24)	12 (27)	4 (11)	1 (4)	4 (25)	3 (37)	1 (10)
Milrinone	0 (0)	9 (3)	9 (11)	0 (0)	0 (0)	0 (0)	0 (0)	0 (0)	0 (0)	0 (0)
Epinephrine	3 (10)	4 (2)	0 (0)	2 (4)	0 (0)	2 (6)	0 (0)	0 (0)	0 (0)	0 (0)
**Endovascular methods to treat symptomatic vasospasm**										
Angioplasty and intra-arterial vasodilator(s)	2 (7)	133 (50)	63 (67)	15 (30)	16 (36)	16 (44)	12 (50)	9 (56)	8 (100)	3 (30)
Intra-arterial vasodilator(s) alone	6 (21)	66 (25)	19 (24)	14 (27)	14 (32)	9 (25)	3 (12)	4 (25)	0 (0)	3 (30)
None of the above	20 (69)	58 (21)	0 (0)	22 (43)	10 (23)	10 (28)	8 (33)	3 (19)	0 (0)	4 (40)
Angioplasty alone	1 (3)	12 (4)	6 (8)	0 (0)	4 (9)	1 (3)	1 (4)	0 (0)	0 (0)	0 (0)
